# Supplementary material for: High diversity in Delta variant across countries revealed by genome‐wide analysis of SARS‐CoV‐2 beyond the Spike protein
Source: Mol Syst Biol. 2022 Feb 14;18(2):e10673. doi: 10.15252/msb.202110673 (PMC8842124; doi:10.15252/msb.202110673)
Supplement: Supplementary file 2 — Appendix [file MSB-18-e10673-s001.pdf]

Appendix Figures and Tables

Table of Contents

Appendix Figure S1 - Probability density of pairwise cosine similarity across countries………..2
Appendix Figure S2 - K-means Sensitivity analyses……………………………………………..3
Appendix Figure S3 - Union set of country-specific core mutations in Spike protein of SARS-CoV-2 variants…………………………………………………………………………...……..…4
Appendix Table S1 - Highly prevalent mutations in the Delta variant……………………………..5
Appendix Table S2 - Computational characterization of country-specific core mutations in the United States ($Delta_{UnitedStates}$) and in India ($Delta_{India}$)...6
Appendix Table S3 - List of the union set of country-specific core mutations unique to the Delta variant's proteome. This table lists the union set of country-specific core mutations (136), across 104 countries, which are unique to the proteome of the Delta variant (as in Fig EV5B)..............8

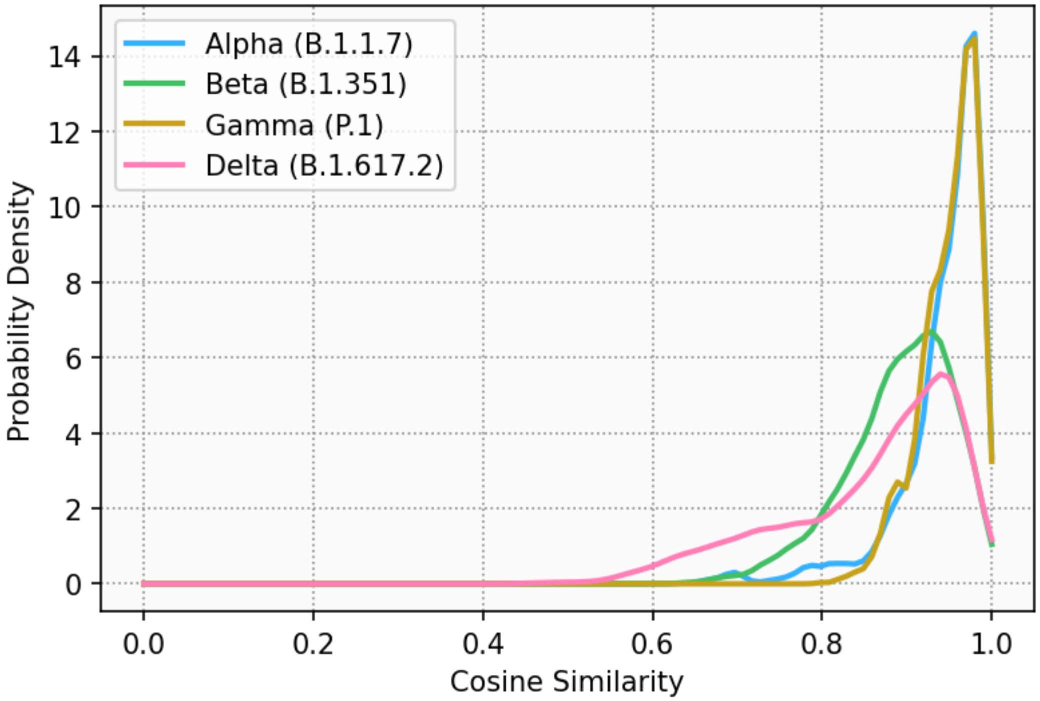

Appendix Figure S1 - Probability density of pairwise cosine similarity across countries. The plot suggests a higher diversity of pairwise cosine similarity values, and thus a higher diversity of the Delta variant across countries.

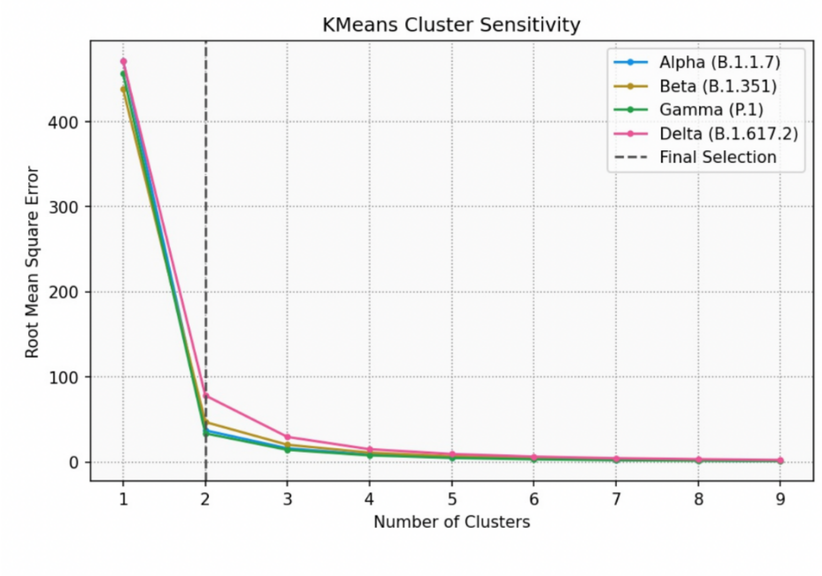

Appendix Figure S2 - K-means Sensitivity analyses. K-means clustering sensitivity analysis. Country-specific core mutations for each variant of concern are calculated using a varying number of clusters (*k*) in the K-means algorithm. For each value of *k*, the Root Mean Square Error (RMSE) was calculated based on the distance of points from the centroid. The mean RMSE across all countries for each value of *k* has been plotted here. The dotted line represents the value of *k* (=2) selected for further analyses.

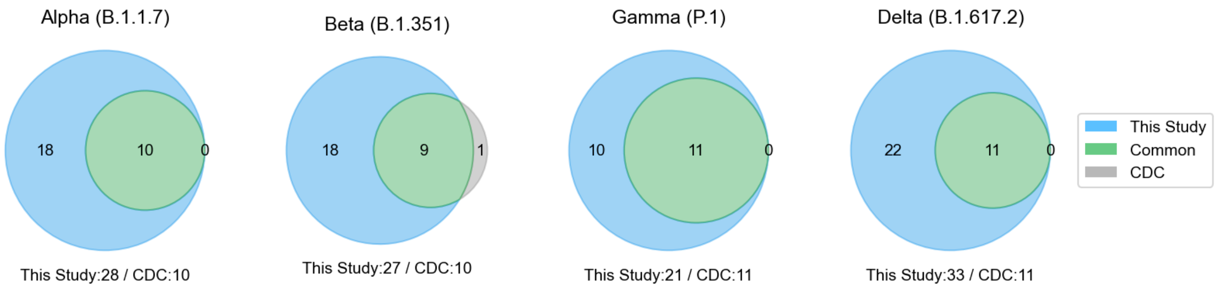

Appendix Figure S3 - Union set of country-specific core mutations in Spike protein of SARS-CoV-2 variants. Comparison of country-specific core Spike mutations versus characteristic Spike mutations. Venn diagrams show a comparison of core Spike mutations identified in this study versus characteristic Spike mutations reported by CDC for each variant of concern. We failed to identify ΔL241 from the CDC's set, which can be attributed to the differential alignment of the deleted positions to the wild-type SARS-CoV-2 genome in the mutation calling pipeline of the genomic assembly. In contrast, we identified multiple mutations in the Spike protein for each lineage that are highly prevalent in one or more countries but are missing from CDC's set of mutations.

Appendix Table S1 - Highly prevalent mutations in the Delta variant. The highly prevalent mutations in the Delta variant are listed here. Other than Spike D614G and NSP12 P323L, all the other mutations are nearly exclusive to the Delta variant.

	**Mean Prevalence (in %)**			
	**Alpha (B.1.1.7)** (n=813,973)	**Beta (B.1.351)** (n=177,46)	**Gamma (P.1)** (n=42,869)	**Delta (B.1.617.2)** (n=198,460)
M I82T	0.03	0.04	0.01	99.90
NS3 S26L	0.15	0.20	0.21	99.92
NS7a T120I	0.15	0.08	0.09	99.72
NS7a V82A	0.01	0.02	0.00	99.38
N D377Y	0.05	0.20	1.00	99.63
N R203M	0.00	0.02	0.00	99.87
NSP12 P323L	99.90	91.68	99.70	99.87
Spike D614G	99.94	99.63	99.97	99.95
Spike L452R	0.05	0.06	0.01	99.85
Spike P681R	0.19	0.03	0.03	99.89
Spike T19R	0.00	0.01	0.00	99.84
Spike T478K	0.01	0.06	0.01	99.88

Appendix Table S2 - Computational characterization of country-specific core mutations in the United States ($Delta_{UnitedStates}$) and in India ($Delta_{India}$). There are 13 mutations that are highly prevalent in $Delta_{UnitedStates}$ but not $Delta_{India}$. In contrast, there are three mutations that are highly prevalent in $Delta_{India}$ but not in $Delta_{UnitedStates}$.

Mutation	Secondary structure	Domain / Site	ConSurf grade*	No. of protein homologs	Overall predicted change in protein function
			**$Delta_{UnitedStates}$**		
Spike G142D	Strand	N-terminal domain[48] (antigenic supersite[37])	1	150 (corona viruses)	Reduced binding of neutralizing antibodies, leading to immune escape[58]
Spike E156G	Helix		8		
Spike ΔF157	Helix		8		
Spike ΔR158	Helix		1		
Nucleocapsid G215C	Helix	-	1	139 (corona viruses)	Increased stability with possibility to form intermolecular disulfide bridge to facilitate dimerization
NSP3 A488S	Loop	Macro2 domain[59]	**	141 (corona viruses)	Altered binding to ADP-ribose, NAD or related ligands[59,60]
NSP3 P1228L	Loop	-	1		Functional impact of the mutation is unclear
NSP4 V167L	Loop	-	1	139 (corona viruses)	Functional impact of the mutation is unclear
NSP4 T492I	Strand	Nsp4C domain[59]	8		Possible effect on interactions with other proteins[36]
NSP6 T77A	Helix	-	7	95 (corona viruses)	Functional impact of the mutation is unclear
NSP14 A394V	Loop	N7-MTase[59] (structurally	1	146	Altered N7-methyltransferase activity, leading to changes in 5′-cap structure of the genome RNA[61]

		proximal to the SAM-binding pocket)		(corona viruses)	
NS7b T40I	Loop	-	-	-	Functional impact of the mutation is unclear
**Delta$_{India}$**					
NSP3 P822L	Helix	Peptidase C16[59]	1	141 (corona viruses)	Functional impact of the mutation is unclear
NSP4 A446V	Helix	Nsp4C domain[59]	4	139 (corona viruses)	Possible effect on interactions with other proteins[36]
NSP6 V149A	Helix	-	1	95 (corona viruses)	Functional impact of the mutation is unclear

* On a scale of 1 (variable) to 9 (conserved)

** Unreliable conservation score due to calculations performed on less than six non-gapped homologous sequences

Appendix Table S3 - List of the union set of country-specific core mutations unique to the Delta variant's proteome. This table lists the union set of country-specific core mutations (136), across 104 countries, which are unique to the proteome of the Delta variant (as in **Fig EV5B**).

Protein	Mutations
Spike (S)	T19R, K77T, T95I, G142D, E156G, ΔE156, ΔF157, R158G, ΔR158, R214H, P251L, D253A, L452R, T478K, E484Q, T572I, D574Y, Q677H, P681R, D950B, D950N, D979E, G1167V, M1229I, D1259Y, V1264L
Nucleocapsid (N)	G18C, D63G, L139F, R203M, G215C, A252S, S327L, D377Y, R385K
Membrane (M)	D3H, I82T
NSP1	E87D, ΔK141, ΔS142, ΔF143
NSP2	K81N, P129L, P200L, P200S, L204F, I251M, A596T
NSP3	A85V, P192L, T422I, A488S, T678I, I707V, H727R, V765F, P778S, P822L, E906D, V1070I, P1103Q, T1189I, L1244F, G1273S, H1274Y, A1311V, P1469S, I1723V, A1736V, T1830I
NSP4	S137L, V167L, T204I, D217N, V293I, C296F, F375S, T492I
NSP5	I213V
NSP8	K37N, L122I
NSP10	A104V
NSP6	A2V, H11Q, T77A, V149A, T181I
NSP12	K91R, M124I, F192V, M196I, R197Q, G228S, D269G, D481A, T644M, G671S, T801I, M818V
NSP13	P77L, V187L, V210I, I334V
NSP14	T16I, P46L, M49I, M72I, A119V, V182I, I363V, A394V, Y420stop
NSP15	K109N, H234Y, R257C, K259R, M330I
NSP16	P215L, R216N, R216C, Q238H
NS3	S92L, A110S, E239D
NS6	K48N
NS8	A65S, S69L, I88T, L95F
NS7a	P45L, V71I, V82A, V104F, L116F, T120I
NS7b	T40I
