# Supplementary material for: High diversity in Delta variant across countries revealed by genome‐wide analysis of SARS‐CoV‐2 beyond the Spike protein
Source: Mol Syst Biol. 2022 Feb 14;18(2):e10673. doi: 10.15252/msb.202110673 (PMC8842124; doi:10.15252/msb.202110673)
Supplement: Supplementary file 3 — Expanded View Figures PDF [file MSB-18-e10673-s002.pdf]

Expanded View Figures

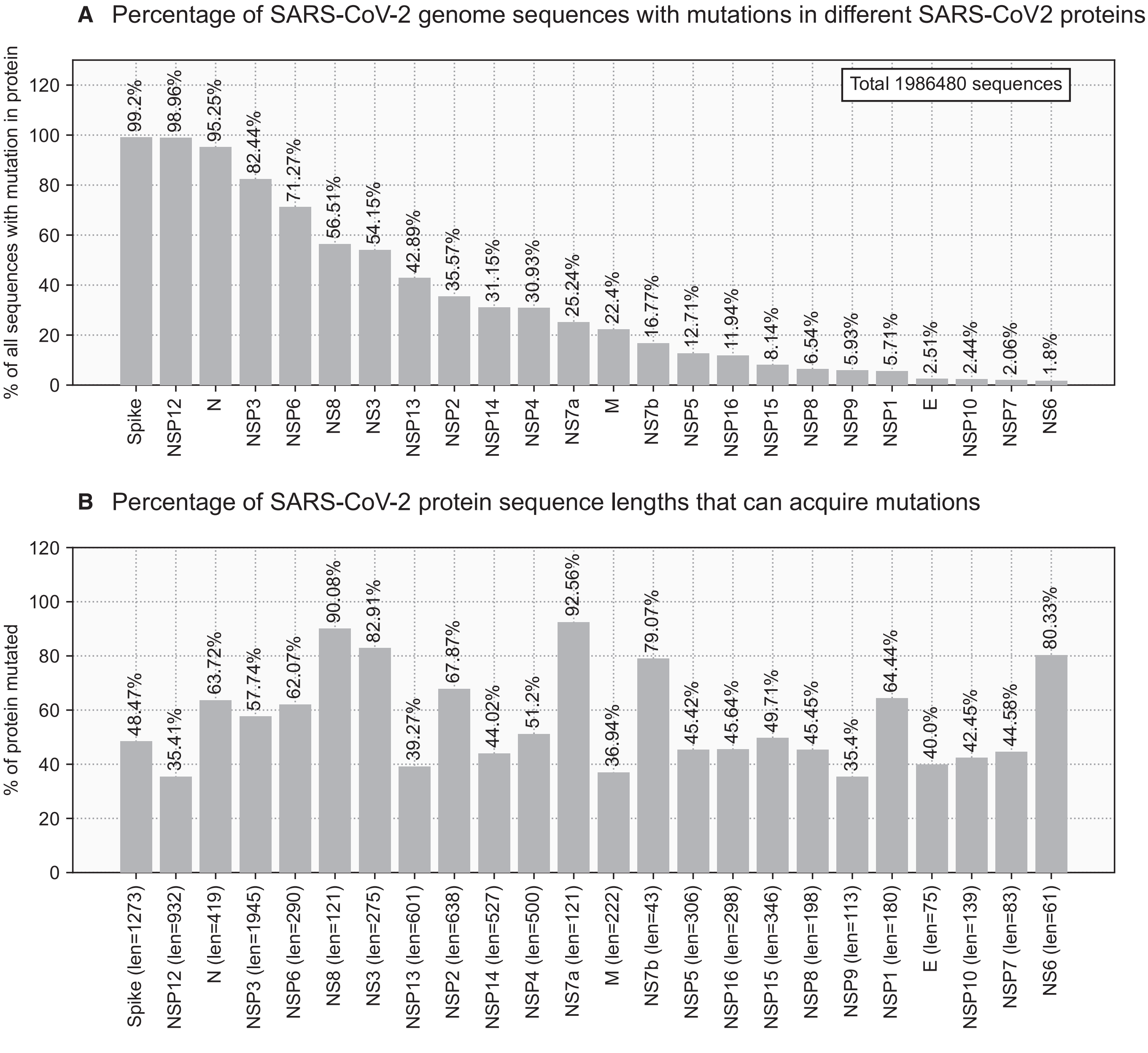

Figure EV1. The proteome-wide mutational profile of SARS-CoV-2.

A Percentage of SARS-CoV-2 genome sequences with mutations in different SARS-CoV-2 proteins. Spike, NSP12, and Nucleocapsid are the most frequently mutated proteins.

B Percentage of SARS-CoV-2 protein sequence lengths that can acquire mutations. The NS7a and NS8 are hypervariable proteins that mutate 92.56% and 90.08% of their sequence lengths. The lengths of the proteins are mentioned in parentheses.

© 2022 The Authors

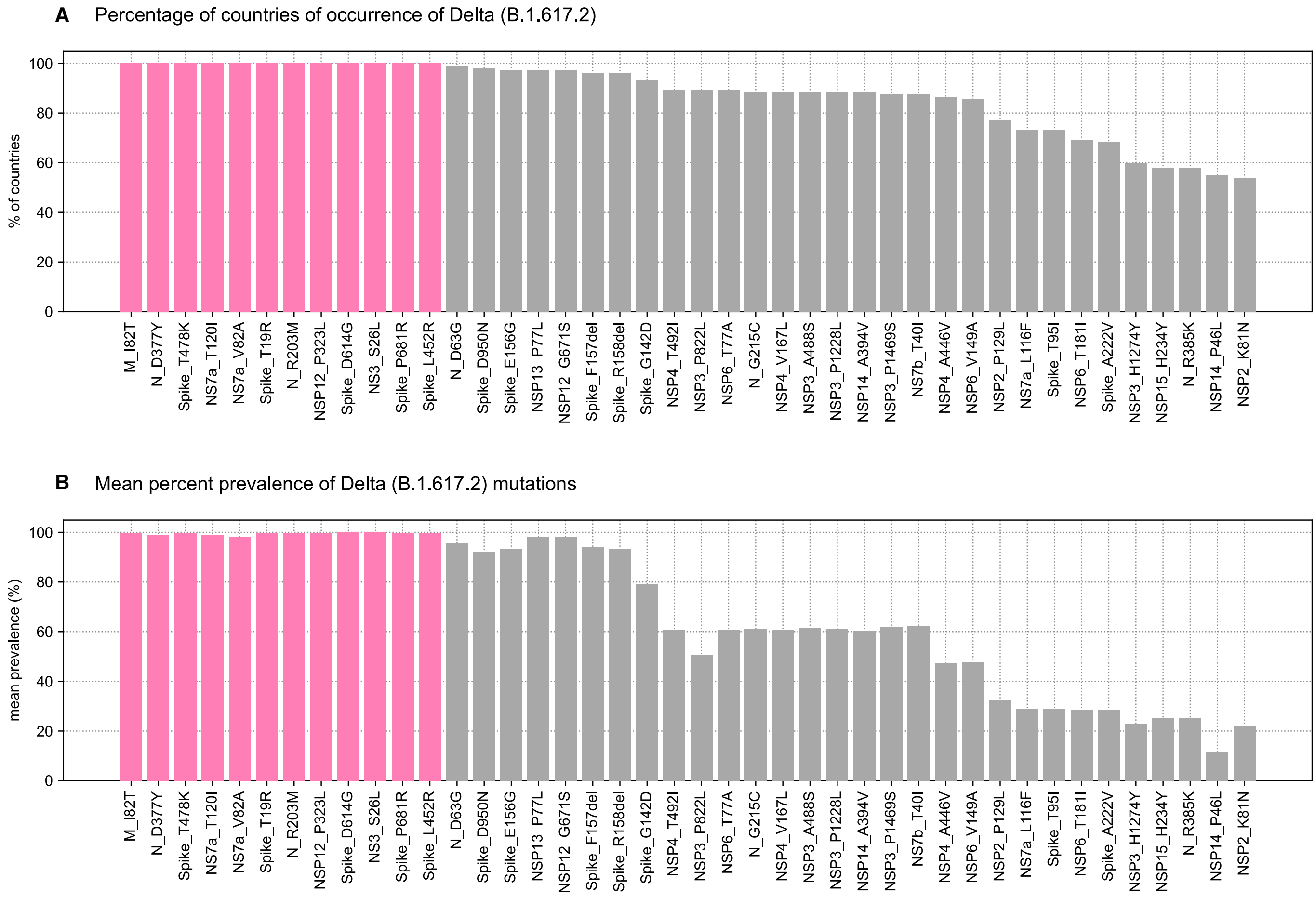

Figure EV2. Prevalence of mutations in the Delta variant.

A Percentage of countries of occurrence of the Delta variant. Only mutations present in 50% or more countries are shown here for representative purposes. Twelve mutations (highlighted in magenta) are reported from all 104 countries where the Delta variant occurs.

B Mean percent prevalence of Delta mutations. The 12 mutations highlighted in magenta are highly prevalent (mean prevalence > 99%) in all countries of occurrence of the Delta variant (Appendix Table S1).

 © 2022 nference, Inc

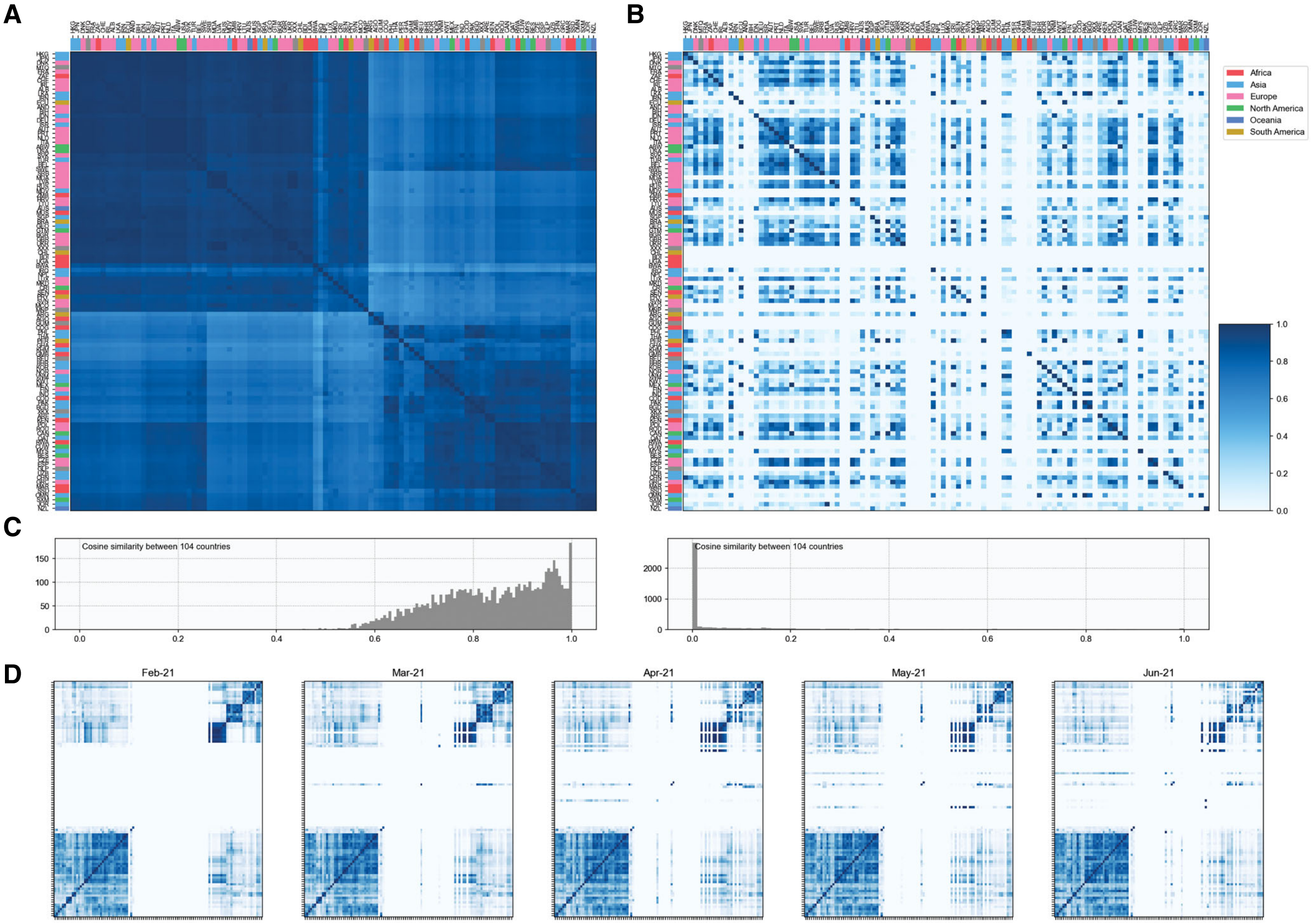

Figure EV3. Effect of geographical separation and airline connectivity on the diversity of the Delta variant.

A Cosine similarity between country-specific core mutations in Delta variant across all countries of its occurrence. This shows that the diversity of the Delta variant is not affected by the geographical separation of the countries.

B Airline connectivity across countries. Sub-variants of the Delta variant may or may not co-exist in countries with good airline connectivity. This shows that the diversity of the Delta variant is not affected by airline connectivity across countries.

C Frequency histogram for the distribution of the cosine similarity values between country-specific core mutations in Delta variant across all countries of its occurrence (left) and airline connectivity across countries (right).

D Temporal airline connectivity. We observe that the patterns of airline connectivity have been unaffected during the period of February 2021 to July 2021.

© 2022 The Authors

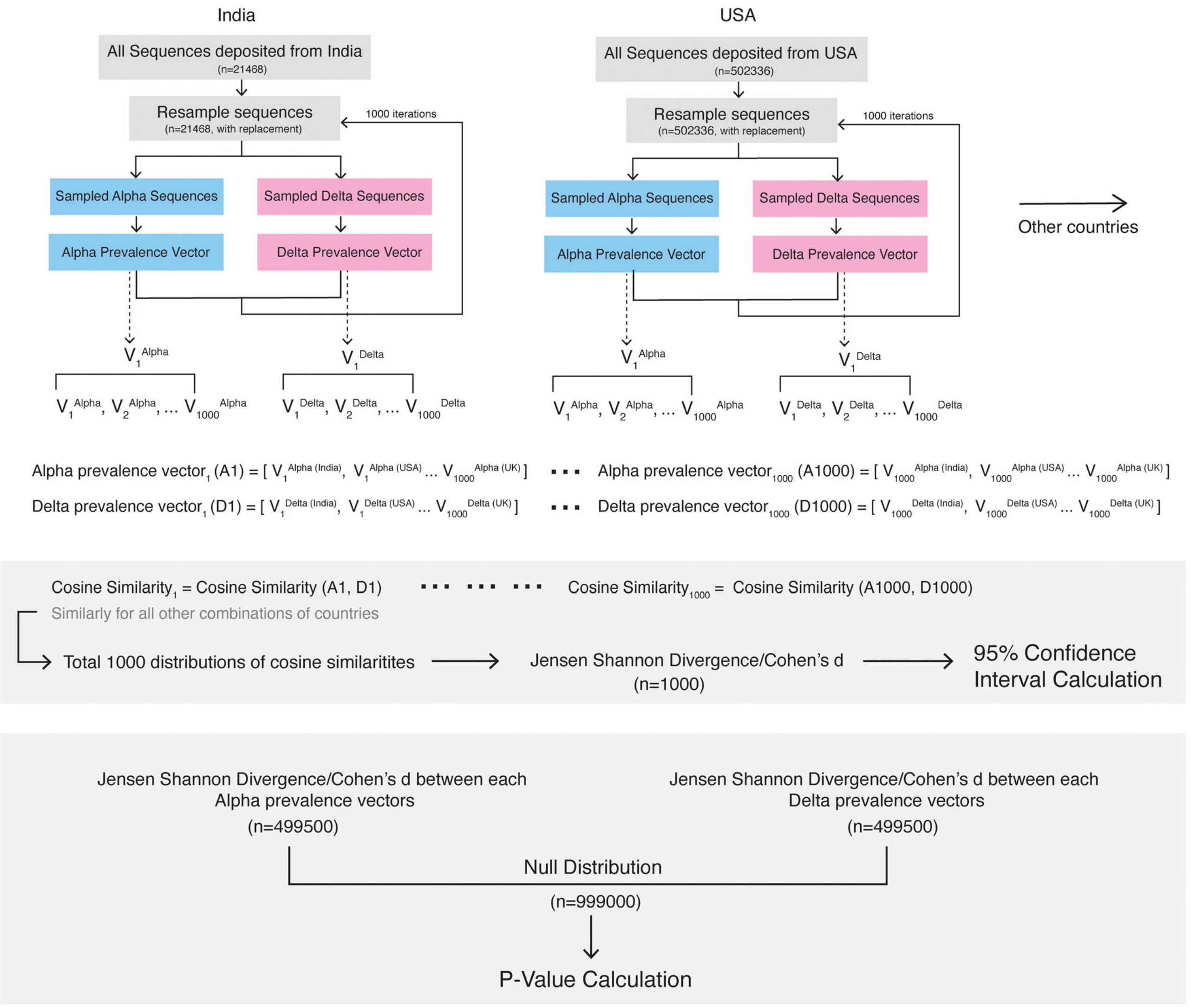

Figure EV4. Bootstrapping Methodology.

For each country, we resampled (with replacement) all the sequences deposited in the GISAID database and generated a cosine similarity distribution for Alpha and Delta variants. For calculating 95% confidence interval, we calculated Jensen–Shannon divergence (JSD) and Cohen's d for each bootstrap iteration. To get a null distribution for JSD and Cohen's d, we calculated these metrics from the Alpha and Delta cosine similarity distribution generated in each bootstrap iteration (n = 1000). The P-values were calculated based on the distribution of all bootstrapped values and original JSD/Cohen's d values.

 © 2022 nference, Inc

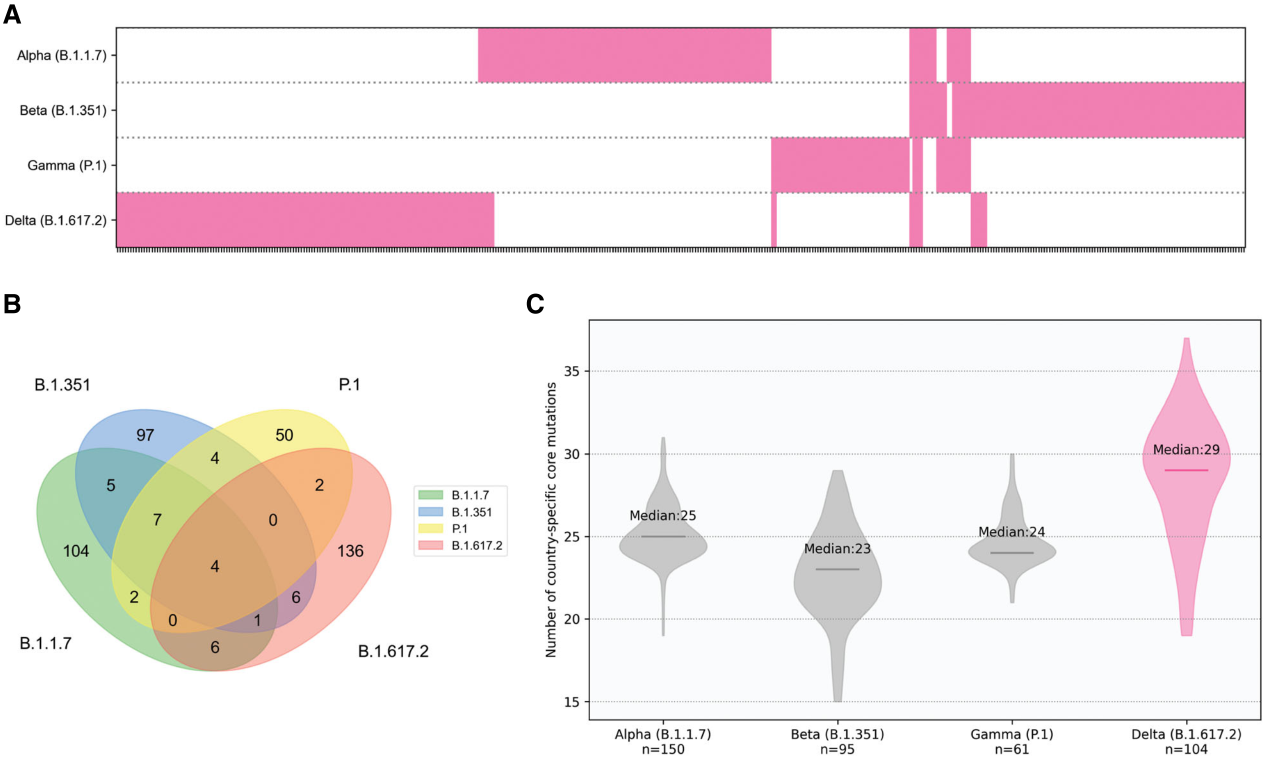

Figure EV5. Distinct mutational profiles of the SARS-CoV-2 variants.

A The different repertoire of mutations in the variants of concern. Each tick on the X-axis represents a mutation and is annotated in magenta if it is a part of the union set of country-specific core mutations for the variant.

B Comparison of union sets of country-specific core mutations across different variants of concern. A total of 136 of 155 (87.74%) mutations in the Delta variant are unique to it.

C Distribution of country-specific core mutation counts across countries for the variants of concern. The median count of country-specific core mutations for each variant is indicated on the plot. The Delta variant has a higher mutational load as compared to the other variants of concern.

© 2022 The Authors
